# Hands

**Published:** 1878-02

**Authors:** 


### HANDS.

The hands are as much abused as the feet, and to know how to serve them properly in winter time may be a convenience and comfort for many. It is a great luxury to go to bed with clean hands, washing them in soap and warm water in winter, so as to dissolve the dust and dirt which settles in the crevices and fills up the pores, thus leaving them soft and pliable; this should be done also in the morning before washing the face, care being taken to rinse them of every particle of soap with clean pure water, and most thoroughly dry them after washing with a soft cloth; otherwise, the skin will soon chap and crack open, especially if gloves are not put on when the fire is stirred or approached for any domestic purpose. The hands should be put in water as seldom as possible in cold weather and if immediately after the washing a teaspoonfull of spirits of wine were rubbed into them or a few drops of honey—the alcohol is preferable—it would greatly tend to prevent chapping altogether. In coming down stairs of a cold day the bannister should not be touched with the hand unless a glove is worn ; if that is not convenient, let the cuff of the sleeve be moved along the bannister if support and guidance is needed in descending the stairs; this may seem a small matter but a lady does not well like having a hand as course as a corn-cob when she holds it out to give her friends a New Year's greeting; besides, chapped hands interfere greatly with domestic requirements, for to say nothing of the painfulness sometimes attending it, it is often impossible to knit or sew with any kind of comfort or convenience. Cold cream, as it is called, tends to prevent and cure chapped hands; and for the convenience of our lady readers who live in the country remote from drug stores, the following directions are appended:

Take of oil of sweet almonds three ounces and a half, one ounce of spermaceti, one hundred and twenty grains of white wax and two ounces or four tablespoonfulls of rosewater; melt the oil, spermaceti and wax in a water-bath, that is in a vessel set in another vessel of boiling water, as a cabinet-makers glue-pot; then add the rose-water gradually, stir it up with a clean stick until it is pretty well cooled, pour it in a tea-cup or other delf ware and rub it into the hands after each washing. Badly chapped hands improve rapidly, if after the night washing common sweet oil is rubbed in thoroughly and a pair of old kid gloves are worn during the night for several nights in succession.

